# The German EMPATHIC-30 Questionnaire Showed Reliability and Convergent Validity for Use in an Intermediary/General Pediatric Cardiology Unit: A Psychometric Evaluation

**DOI:** 10.3389/fcvm.2022.901260

**Published:** 2022-06-23

**Authors:** Alona Girch, Ralph C. A. Rippe, Jos M. Latour, Michaela Jönebratt Stocker, Magdalena Blendermann, Katharina Hoffmann, Hannes Heppner, Felix Berger, Katharina R. L. Schmitt, Hannah Ferentzi

**Affiliations:** ^1^Department of Pediatric Cardiology, German Heart Center Berlin, Berlin, Germany; ^2^Charité University Hospital Berlin, Berlin, Germany; ^3^Research Methods and Statistics, Institute of Education and Child Studies, Leiden University, Leiden, Netherlands; ^4^School of Nursing and Midwifery, Faculty of Health, University of Plymouth, Plymouth, United Kingdom; ^5^Department of Psychology, University of Montana, Missoula, MT, United States

**Keywords:** congenital heart disease, family-centered care, pediatric cardiology, psychometric properties, internal consistency reliability, convergent validity, construct validity

## Abstract

**Background:**

Family-Centered Care is a useful framework for improving care for hospitalized children with congenital heart disease. The EMpowerment of PArents in THe Intensive Care-30 (EMPATHIC-30) questionnaire is a widely accepted tool to measure parental satisfaction with Family-Centered Care. Psychometric properties of the EMPATHIC-30 have been evaluated in neonatal and pediatric intensive care units, but not in pediatric cardiac care units. Therefore, our aim was to assess the psychometric properties of the German EMPATHIC-30 in an intermediary/general pediatric cardiology unit.

**Methods:**

We used data from a quality management survey comprising the German EMPATHIC-30, a sociodemographic questionnaire and four general satisfaction items. Data were collected at the intermediary/general pediatric cardiology unit of a specialized heart center in Germany (*n* = 366). We split the data randomly into two subsets. In the first subset, we assessed internal consistency reliability with McDonald's omega and Cronbach's alpha, and convergent validity using Spearman's rank correlation. Furthermore, we explored the internal structure with Principal Component Analysis (PCA). In the second subset, we validated the resulting structure using Confirmatory Factor Analysis (CFA).

**Results:**

The reliability estimates exceeded 0.70 for all five domain scores and 0.90 for the full-scale score. Convergent validity between EMPATHIC-30 domain scores/ the full-scale score and the four general satisfaction items was adequate (r_s_ = 0.40–0.74). The PCA suggested three components, accounting for 56.8% of the total variance. Cross-validation via CFA showed poor model fit (χ^2^ = 1545.78, χ^2^/df = 3.85, CFI = 0.70, TLI = 0.66, RMSEA = 0.13), indicating that the EMPATHIC-30 shows no clear and generalizable factor structure in this sample.

**Discussion:**

The German version of the EMPATHIC-30 exhibited reasonable psychometric properties in an intermediary/general pediatric cardiology unit. Follow-up studies should investigate the factor structure of the EMPATHIC-30 in other pediatric inpatient care settings.

## Introduction

Congenital Heart Disease (CHD) is defined as a structural defect of the heart or intrathoracic vessels (1). With a global prevalence of 9.41 per 1,000 births, it represents the most common birth defect worldwide (2, 3). In Europe, ~36 000 children are born with a CHD each year and around 28% of them have moderate to complex heart defects, requiring interventional or surgical treatment (4). During hospitalization, they are exposed to a myriad of stressors, such as separation from their parents, a stressful environment with bright lights and loud noises, restricted mobility, and disrupted sleep. Research shows that children with CHD are at risk for neurodevelopmental impairment, as well as emotional, social, and behavioral difficulties (5–7). Distress during hospitalization may contribute to these challenges (7, 8). Hence, optimizing the hospital environment potentially is an effective strategy to improve neurodevelopmental and psychosocial outcomes of children with CHD, for which Family-Centered Care (FCC) provides a useful framework (9).

Family-Centered Care is an international standard of healthcare provision based on a mutually beneficial partnership among the healthcare providers, patients, and their families (10, 11). In pediatrics, FCC emphasizes the parents as their child's primary source of emotional, social, and developmental support and acknowledges them as integral part of the healthcare team (12). Specific FCC interventions either target the parents (e.g., educational programs, participation of parents in medical rounds), the parent-child dyad (e.g., promoting skin-to-skin contact), or the health-care ecosystem as a whole (e.g., structural implementation of a primary nursing model) (13). Most studies investigating the effects of FCC interventions on child and parent wellbeing have been conducted in Neonatal Intensive Care Units (NICUs), with positive effects reported for physical wellbeing, stress regulation, sleep, and neurodevelopmental outcomes of the child, parent-child attachment, and parental mental wellbeing (14–17). A meta-analysis of randomized controlled trials showed that FCC interventions improve physical health outcomes in premature infants (e.g., weight gain), while their parents experience less anxiety, depression, and stress (18). Despite the positive effects of FCC interventions in neonatology, studies investigating FCC in children with CHD are scarce. However, several authors argue that FCC practices may be similarly beneficial in this population (19–21).

Measuring the subjective experience of provided care is crucial for advances in this area of research, especially when FCC principles are not structurally implemented yet (22). In order to measure parent satisfaction with FCC, the EMpowerment of PArents in THe Intensive Care (EMPATHIC) questionnaire is frequently used (23). Latour et al. (24, 25) originally developed the questionnaire for Pediatric Intensive Care Units (PICUs), based on expert opinions from over 300 PICU nurses and physicians, as well as over 600 parents of children discharged from a PICU. The original scale comprises 65 items, with each item reflecting care aspects from one of the following five domains: Information, Organization, Parental Participation, Care and Cure, and Professional Attitude (23). The domains were identified in qualitative analyses and evaluated quantitatively, by using Confirmatory Factor Analysis (CFA), with separate models for each domain. The authors subsequently developed a shortened version of the questionnaire, the EMPATHIC-30, to improve user friendliness (26). The number of items was reduced by means of multiple regression analysis, resulting in 30 items. In the past years, the EMPATHIC-30 gained international popularity and has been translated from Dutch into various languages, including English, Spanish, Turkish, and German (27–30).

In the original publication of the EMPATHIC-30, Latour et al. (26) found high internal consistency reliability estimates for the five domain scores and the full-scale score. Gill et al. (27) tested the questionnaire's psychometric properties in Australian PICUs, NICUs, and general pediatric wards and reported similar values for the internal consistency reliability (27). Above that, the questionnaire showed adequate convergent validity, as assessed by moderate to strong correlations between each of the domain scores and four general satisfaction items, pointing toward applicability of the questionnaire in these care settings. Orive et al. (28) investigated internal consistency reliability and convergent validity of the questionnaire in Spanish PICUs, with similar results. Only few studies have investigated the construct validity of the questionnaire by using factor analysis. Factor analysis is a statistical method to identify latent variables, which explain covariation amongst a set of measured variables (31). It is therefore an essential approach to generate and evaluate hypotheses about the underlying construct an instrument aims to measure (32). Tiryaki et al. (29) investigated psychometric properties of the EMPATHIC-30 in Turkish NICUs and conducted a CFA in a sample of 238 parents. The authors found a moderate model fit of the final factor solution. However, the factor structure was not reported and thus remains unclear. The German version of the EMPATHIC-30 has not been evaluated psychometrically (30). Furthermore, while the EMPATHIC-30 has been extensively evaluated in different care settings, it has not been psychometrically tested for use in pediatric cardiology units.

Therefore, our aim was to evaluate the psychometric properties, specifically internal consistency reliability, convergent validity, and factor structure of the German EMPATHIC-30 at an intermediary/general pediatric cardiology unit. In order to assess internal consistency reliability, we used McDonald's omega. Although controversially discussed in the literature, we additionally present the classical Cronbach's alpha, to allow for direct comparison to other studies (33, 34). To assess convergent validity, we investigated the relationship between the domain scores and the full-scale score with four general satisfaction items, comparable to the methodology of above-mentioned studies. Furthermore, we investigated the factor structure of the questionnaire, following a two-step procedure. In the first step, we explored the internal structure of the questionnaire on half of the data using Principal Component Analysis (PCA) rather than Exploratory Factor Analysis (EFA). While both PCA and EFA are variable reduction techniques, EFA assumes an underlying construct, which is not measured directly, and PCA reflects a linear combination of variables. We used PCA to explore the internal structure of the questionnaire, because our focus was to explore the structure in total item variance including error, without making assumptions on latent constructs, as these were unknown for the current context (35). In a second step, we used three separate CFA on the other half of the data: The first CFA was conducted to validate the structure resulting from the PCA. The second CFA was conducted to investigate a one-component solution, motivated by potential unidimensionality of the scale. The third CFA was conducted to investigate a five-component solution motivated by the five domains of the EMPATHIC-30.

## Materials and Methods

### Study Design and Setting

For the psychometric evaluation of the EMPATHIC-30 questionnaire, we used data from a quality management survey comprising the German EMPATHIC-30, a socio-demographic questionnaire, four general satisfaction items and open commentary fields. Data were collected at the intermediary/general pediatric cardiology unit of the German Heart Center Berlin. With its 24 monitored beds and 1,200 yearly admissions, the unit provides specialized care to patients of all ages, ranging from infants to adults, with varying degrees of CHD. This study was approved by the Medical Ethics Committee Charité Virchow (Nr EA2/032/20).

### Procedures

All parents of children with CHD hospitalized at the ward were invited to participate in the quality management survey. Participation was voluntary and anonymous. At discharge, doctors handed out a paper and pencil version of the survey together with a return envelope. After completing the survey, parents returned it in a mailbox on the ward. Data collection took place between August 2019 and June 2021.

### Materials

The German EMPATHIC-30 questionnaire comprises 30 statements spanning five domains: Information (5 items), Organization (5 items), Parental Participation (6 items), Care and Cure (8 items), and Professional Attitude (6 items). Every statement is rated on a six-point scoring-scale ranging from 1 “certainly no” to 6 “certainly yes,” or rated 0 for the answer alternative “not applicable.”

Sociodemographic information was obtained through a purpose-designed questionnaire. It contains one item to specify the respondent (with options “mother,” “father,” “both mother and father,” and “other relatives” with the option of open-ended specification), as well as items relating to age of the child, place of birth and mother tongue of the parents, length of hospital stay, type of and reason for admission, and undertaken medical procedures.

Four general satisfaction items were included in the survey: Two items are rated on the same six-point scale as the EMPATHIC-30 questionnaire: “We would recommend this unit or ward,” “We would be happy to return to this unit or ward”. Two more items are rated on a ten-point scale, ranging from “very bad” to “excellent”: “Overall performance of doctors” as well as “Overall performance of nurses” (23). Furthermore, commentary fields were included in the survey about general experiences made during admission, hospital stay, and discharge.

### Statistical Analyses

Statistical analyses were carried out using SPSS 27 (SPSS Inc, Chicago, Illinois). Non-linear and linear PCA were conducted in SPSS. AMOS, an SPSS extension module, was used for the CFA.

#### Data Preparation and Preliminary Analyses

##### Handling of Answer Alternative “Not Applicable”

Non-linear Principal Component Analysis (CATPCA) was performed to determine the best linear replacement values for observed scores in each item individually, for the scores 0 up to 6 (0 corresponding to the answer alternative “not applicable”) (36). Based on transformation plots from nominal optimal scaling, the scores 0 and 6 got assigned a similar quantification; both answer categories had an equivalent interpretation by participants. This was consistent with previous findings by Latour et al. (23). Scores on the answer category “not applicable” were therefore recoded to the highest value of the scale (i.e., 6). In addition, the transformation plots revealed that the answer categories functioned as near-equally spaced linear scale; models with nominal transformation and with numerical transformation after recoding yielded 0.8% difference in explained variance. All subsequent linear analyses were performed using the recoded scores.

##### Handling of Missing Data

Returned questionnaires with ≥75% of missing items were excluded from analysis. One third of respondents presented at least one missing value and the total percentage of missing data points was 2.3%. Missing data can affect the estimation and interpretation of PCA (37). Little's Missing Completely at Random (MCAR) test was significant, indicating that missings are not missing completely at random, thus indicating a potentially systematic difference between missing and observed values (38). Therefore, multiple imputation, a proven statistical method to estimate missing values, was used on the recoded scores. Missing scores were estimated in 25 sets, applying Markov Chain Monte Carlo sampling and predictive mean matching (39). Results of the statistical analyses were pooled for the imputed data sets whenever possible.

##### Data Split for Separate Estimation and Validation

The data set was randomly split in half, creating two subsets (A, B) to perform 2-fold cross-validation. All statistical structure and content analyses were performed on set A. Set B was used only as validation set for the confirmatory evaluation of the internal structure via CFA.

#### Descriptive Statistics

Descriptive statistics of the EMPATHIC-30 scores as well as sociodemographic characteristics of the sample are reported (means and standard deviations for quantitative variables, absolute frequencies and percentages for categorical variables). To check for successful randomization, descriptive statistics for the full set, analysis set A, and validation set B, as well as test statistics for the comparison between set A and B are provided.

#### Internal Consistency Reliability

The internal consistency reliability of the German EMPATHIC-30 questionnaire on domain and full-scale level was assessed with McDonald's omega. Cronbach's alpha was computed additionally. Values greater than 0.70, 0.80, and 0.90 reflect acceptable, good, and excellent reliability, respectively (40).

#### Convergent Validity

To examine convergent validity of the questionnaire, we used Spearman's rank correlation test for non-normally distributed data, as assessed visually and through significant Shapiro Wilk tests (*p* < 0.01). We assessed the relationship between the domain scores/ the full-scale score and the four overall satisfaction statements. Based on findings from other validation studies, we expected moderate to strong correlation coefficients, ranging from 0.40 up to 0.79, indicating adequate convergent validity (41).

#### Internal Structure

##### Principal Component Analysis

We conducted a PCA to explore the internal structure of the questionnaire. An oblique rotation should be applied, which reorients the components in order to simplify the mathematical model and interpretation by allowing for intercorrelations between the components. However, this rotation is not implemented for multiply imputed data. Therefore, we conducted a two-step procedure. First, we performed a PCA on the unimputed data set A to determine the number of components. Pairwise deletion was selected to handle missing values. The suitability of the data was assessed with the Kaiser-Meyer-Olkin (KMO) measure of sampling adequacy and Bartlett's test of sphericity. In this exploratory stage, the KMO value is interpreted as an approximation of the ratio of potential common variance compared to the total variance in the data and thus provides information if subsequent factor analysis is suitable. Final component extraction was based on the combined Monte Carlo Parallel Analysis and examination of the scree plot (42). Oblique rotation allowing for intercorrelations between the components was applied in this step. For items with cross-loadings, the component on which the item loaded higher was selected. Loadings under 0.30 (<10% shared variance between item and component) were considered as negligible and therefore not considered for inclusion in the component structure. Second, we used the results of this PCA to motivate the number of components in a second PCA on the imputed data set A by using Generalized Procrustes Analyses in the subroutine by Wingerde et al. (43). This subroutine imposed a pre-specified number of components and orthogonal rotation of the component loadings, ignoring intercorrelations between the components.

##### Confirmatory Factor Analysis

We conducted three separate factor analyses on set B of the sample. First, we conducted a CFA to validate the component structure resulting from the two-step PCA. Second, we conducted a CFA based on a one-component model to investigate potential unidimensionality of the questionnaire. Third, we conducted a CFA based on a five-component model to investigate the validity of the five domains of the EMPATHIC-30 (Information, Organization, Parental Participation, Care and Cure, and Professional Attitude). In the CFA measurement models, correlation between the components is allowed. As combining the results of multiply imputed data is not possible in AMOS, we conducted the analyses on the data with missing values using Full Information Maximum Likelihood Estimation and compared model estimates for robustness. To assess model fit, we used the following fit indices: model-Chi-squared test divided by the degrees of freedom (χ^2^/df), Comparative Fit Index (CFI), Tucker Lewis Index (TLI), and Root Mean Square Error of Approximation (RMSEA). Cut-off values were: χ^2^/df <3, CFI of at least 0.90, TLI of at least 0.95, and RMSEA <0.08 (44, 45). A second evaluation of robustness of findings was performed by repeating the same analyses on the other half of the data (set A).

## Results

A total of 475 questionnaires were returned between August 2019 and June 2021. The response rate was 68% (percentage of returned questionnaires vs. distributed copies). To ensure homogeneity of the data set, we only included questionnaires filled out by parents. As a result, we excluded 91 questionnaires filled out by adult patients, as well as nine questionnaires filled out by relatives other than parents. Upon first exploration of data, we excluded three more questionnaires with comments in the commentary fields reflecting very high satisfaction, but with lowest possible scores on EMPATHIC-30 items, potentially indicating a mix up between highest and lowest scores. Above that, we excluded six questionnaires with ≥75% missing items. The final number of questionnaires included in the analysis was 366, resulting in 183 questionnaires each for analysis set A and validation set B.

### Descriptive Statistics

The child and parent characteristics are presented in Table 1. No significant differences between set A and set B were observed for any of the characteristics, except for the item specifying the respondent, in which a significant shift of mother-only to both parents was seen (X_2__(2, 356)_ = 8.17, *p* = 0.017). As the proportion of mothers giving their input does not differ in both sets, we view this difference as negligible. Therefore, we consider the reported characteristics of each set representative for the whole group. Below, we present the characteristics of set A, as this set drives the main psychometric analysis. Most children of participating families were either infants (*n* = 53, 29.6%), toddlers (*n* = 30, 16.8%) or preschoolers (*n* = 39, 21.8%) and the mean age was 5.32 years (SD = 6.63). Seventy-six percent of the questionnaires were completed by mothers. The majority of participants were born in Germany (*n* = 166, 91.2%) and native German speakers (*n* = 148, 83.1%). Only 7% of hospital admissions were unexpected and the mean length of hospital stay was 6.32 days (SD = 8.86), ranging from 1 to 105 days.

**Table 1:** Characteristics of children and parents in set A, B, and the full sample.

**Characteristics**	**Set A**		**Set B**		**Full sample**		***P*** **-value**
	***n***	**%**	***n***	**%**	***n***	**%**	
**Questionnaire completed by**							
Mother	136	76.4	15	64.6	251	70.5	0.017^a^*
Father	23	12.9	25	14	48	3.5	
Both	19	10.7	38	21.3	57	15.6	
**Country of birth**							
Germany	166	91.2	154	84.2	320	87.7	0.093^a^
Other	15	8.2	25	13.7	40	11	
Both^1^	1	0.5	4	2.2	5	1.4	
**Mother tongue**							
German	148	83.1	143	79	291	81.1	0.548^a^
Other	24	13.5	32	17.7	56	15.6	
Both^1^	6	3.4	6	3.3	12	3.3	
**First admission**							
Yes	95	52.5	99	54	194	53.6	0.673^a^
No	86	47.5	82	45.3	168	46.4	
**Type of admission**							
Planned	165	91.7	174	97.2	339	94.4	0.055^a^
Unexpected	13	7.2	5	2.8	18	5	
Both	2	1.1	0		2	0.6	
**Medical procedures** ^**2**^							
Cardiac surgery	88		103		191		0.154^a^
Heart catheterization	93		79		172		0.102^a^
Medication	19		20		39		0.851^a^
Other	13		13		26		0.988^a^
**Age of the child in years**							
Mean (SD)	5.32 (6.63)		4.98 (6.25)		5.15 (6.43)		0.589^b^
**Length of stay in days**							
Mean (SD)	6.32 (8.86)		7.92 (24.15)		7.13 (18.28)		0.784^b^

SD, standard deviation. Total number of respondents vary because of missing data. ^1^Different answer for either parent when questionnaire filled out by both. ^2^Multiple answers were possible. ^a^Pearson's Chi-square test. ^b^Mann-Whitney U-test. * Statistically significant (p < 0.05).

Parents gave high ratings on the EMPATHIC-30 and all except four items showed mean scores above 5 (Table 2). On the domain level, mean scores ranged from 5.19 (SD = 0.84) for the domain Organization up to 5.45 (SD = 0.76) for the domain Professional Attitude. The “not applicable” response was given most frequently for the item “The unit could easily be reached by telephone” (*n* = 42, 23%).

**Table 2:** EMPATHIC-30 means and standard deviations for set A, B and the full sample.

**Items**	**Set A**		**Set B**		**Full sample**	
	**Mean**	**SD**	**Mean**	**SD**	**Mean**	**SD**
**Information**
Disease treatment	5.52	0.97	5.49	0.97	5.50	0.97
Examination	5.49	0.92	5.56	0.79	5.52	0.86
Drugs	4.99	1.23	5.19	1.13	5.09	1.19
Daily talks with doctor	5.37	1.07	5.46	1.11	5.41	1.09
Daily talks with nurse	5.59	0.94	5.63	0.99	5.61	0.97
**Organization**
Clean	5.55	0.89	5.64	0.78	5.59	0.84
Reachable	5.75	0.65	5.80	0.61	5.78	0.63
Noise	4.94	1.27	5.09	1.21	5.02	1.24
Space	4.70	1.51	4.83	1.47	4.76	1.47
Efficiency	5.09	1.20	5.13	1.22	5.11	1.21
**Parental participation**
Decision-making	5.20	1.17	5.30	1.13	5.25	1.15
Encouraged to stay close	5.22	1.18	5.24	1.30	5.23	1.24
Stay close	5.55	0.92	5.59	0.96	5.57	0.94
Asked about experiences	4.75	1.51	4.60	1.67	4.68	1.59
Confidence in doctor	5.65	0.84	5.73	0.75	5.69	0.79
Confidence in nurse	5.59	0.87	5.67	0.76	5.63	0.82
**Care and cure**
Teamwork	5.45	0.93	5.46	0.87	5.45	0.90
Pain treatment	5.57	0.91	5.57	0.88	5.57	0.90
Child comfort doctor	5.49	0.91	5.51	0.95	5.50	0.93
Child comfort nurse	5.65	0.76	5.61	0.84	5.63	0.80
Responsible doctor	5.00	1.49	5.12	1.38	5.06	1.43
Responsible nurse	5.41	1.25	5.44	1.09	5.43	1.17
Discharge doctor	5.27	1.25	5.37	1.21	5.32	1.23
Discharge nurse	5.34	1.15	5.44	1.11	5.39	1.13
**Professional attitude**
Admission	5.42	0.95	5.26	1.14	5.34	1.05
Hygiene	5.57	0.90	5.60	0.88	5.59	0.89
Privacy	5.07	1.26	5.12	1.21	5.10	1.23
Respect	5.56	0.86	5.65	0.85	5.61	0.86
Sympathy doctor	5.54	0.94	5.61	0.88	5.58	0.91
Sympathy nurse	5.56	0.94	5.64	0.84	5.60	0.89
**General satisfaction items**
Recommend ward	5.56	0.87	5.56	0.89	5.56	0.88
Readmission to ward	5.57	0.90	5.54	0.98	5.56	0.94
Overall rating doctor	9.06	1.49	9.12	1.63	9.09	1.56
Overall rating nurse	9.07	1.44	9.00	1.62	9.03	1.53

*SD, standard deviation. Descriptive statistics were computed on the unimputed data. The answer category 0 (“not applicable”) was treated as missing value*.

### Internal Consistency Reliability

McDonald's omega on the domain level ranged from 0.75 (Organization) to 0.87 (Professional Attitude; Care and Cure) and reached 0.95 for the full-scale. Cronbach's alpha on the domain level was only slightly lower and ranged from 0.73 (Organization) to 0.85 (Professional Attitude). The findings are presented in Table 3.

**Table 3:** Internal consistency reliability of the EMPATHIC-30 domain scores and full-scale score (*n* = 178).

	**Mean (SD)**	**Cronbach's alpha,** **mean over imputed data sets (range)**	**McDonald's omega,** **mean over imputed data sets (range)**
**Domain scores**
Information	5.41 (0.75)	0.78 (0.77–0.79)	0.80 (0.79–0.80)
Organization	5.19 (0.84)	0.73 (0.72–0.73)	0.75 (0.75–0.76)
Parental participation	5.34 (0.82)	0.83 (0.81–0.84)	0.84 (0.83–0.85)
Care and cure	5.49 (0.80)	0.85 (0.84–0.85)	0.87 (0.86–0.87)
Professional attitude	5.45 (0.76)	0.85 (0.85–0.86)	0.87 (0.86–0.87)
**Full-scale score**	5.36 (0.70)	0.95 (0.95–0.95)	0.95 (0.95–0.95)

*SD, standard deviation*.

### Convergent Validity

As shown in Table 4, the correlations between the EMPATHIC-30 domain scores and scores on the four overall satisfaction statements ranged from r_s(183)_ = 0.40, *p* < 0.01 between the domain Organization and satisfaction statement “Readmission to ward,” to r_s(183)_ = 0.68, *p* < 0.01 between the domain Care and Cure and satisfaction statement “Overall rating doctors.” The lowest correlations were found for the domain Organization, with correlations under 0.50 for all satisfaction statements. Similarly, the correlations between the full-scale score and scores on the four overall satisfaction statements ranged from r_s(183)_ = 0.62, *p* < 0.01 for the statement “Readmission to ward” to r_s(183)_ = 0.74, *p* < 0.01 for the statement “Overall rating doctors.” All correlations were significant and moderate to high, according to expectation. For an overview of correlations between the domain scores, see Table 5.

**Table 4:** Spearman's rank correlation coefficient between domain scores/ full-scale score and scores on the overall satisfaction items (*n* = 183).

	**Recommend** **ward**	**Readmission** **to ward**	**Overall rating** **doctors**	**Overall** **rating nurses**
**Domain scores**
Information	0.57	0.53	0.60	0.54
Organization	0.43	0.40	0.46	0.41
Parental participation	0.62	0.58	0.63	0.54
Care and cure	0.58	0.56	0.68	0.67
Professional attitude	0.63	0.57	0.64	0.61
**Full-scale score**	0.67	0.62	0.74	0.69

*All correlations significant (p < 0.01), two-tailed*.

**Table 5:** Spearman's rank correlation coefficient between domain scores (*n* = 183).

	**Information**	**Organization**	**Parental** **participation**	**Care** **and cure**	**Professional** **attitude**
Information	1.00				
Organization	0.49	1.00			
Parental participation	0.69	0.52	1.00		
Care and cure	0.70	0.52	0.70	1.00	
Professional attitude	0.63	0.52	0.67	0.69	1.00

*All correlations significant (p < 0.01), two-tailed*.

### Internal Structure

#### Principal Component Analysis

**Figure 1:**
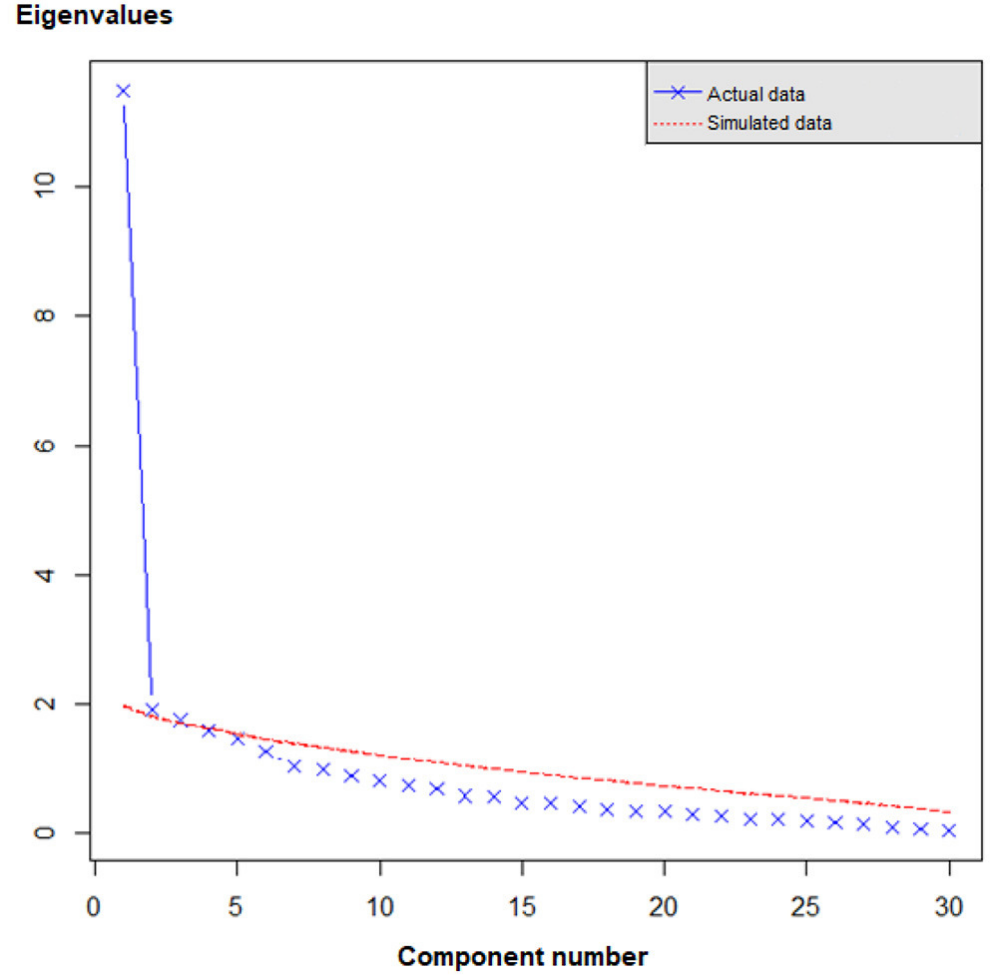
Scree plot. Actual data: unimputed complete case observed data from set A. Simulated data: simulated random data from Monte Carlo Parallel Analysis. The strongest inflection in the empirical data is observed after the first component, suggesting unidimensionality. The lines cross between component 3 and 4, indicating that three *components* based on actual observed data provided more substantive information compared to three components based *on purely random* data. Four or more components should therefore not be extracted from the observed data.

For the first PCA on unimputed data, sampling adequacy was ascertained by a KMO value of 0.89 and a significant Bartlett's test of sphericity (χ^2^ = 3734.43, *p* < 0.01). The comparison of empirical data to simulated random data through Monte Carlo Parallel Analysis suggested a three-component solution, explaining 56.8% of the total variance, with each component accounting for the following percentages: 43.7, 7.5, and 5.6%. In the scree plot (Figure 1), we observed the strongest inflection after the first component, which visually supports unidimensionality of the scale. The obliquely rotated component loadings for the three-component solution based on the first PCA are presented in the Supplementary Table. The correlations between the components were ranging from 0.28 to 0.46. The orthogonally rotated combined component loadings from the imputed data in the second PCA showed a similar three-component solution; except for two items (Professional Attitude - Admission, Organization - Efficiency) all of them loaded on the same respective components. Both items showed strong associations with more than one component in either version of the PCA and seemed to contribute mainly to the intercorrelations among the components. The comparison between component loadings for the first, oblique PCA and second, orthogonal PCA are presented in the Supplementary Table, found in the Supplementary Material.

#### Confirmatory Factor Analysis

The first CFA was conducted to validate the fit of the three-component solution. As the results of both alternative PCAs showed a comparable three-component solution, we chose to start with a CFA model based on results from the second PCA (on imputed data with orthogonal rotation). However, model fit was poor (χ^2^ = 1545.78, χ^2^/df = 3.85, CFI = 0.70, TLI = 0.66, RMSEA = 0.13). The model was then further revised using Lagrange Multiplier Tests (LM test), evaluating several alterations aiming at reducing large correlation residuals. The best fitting solution was found after first removing four items and then applying LM test improvements, yet overall model fit remained poor (χ^2^ = 1640.05, χ^2^/df = 5.05, CFI = 0.73, TLI = 0.70, RMSEA = 0.09).

The alternative model specifying pure unidimensionality showed poor goodness of fit, with χ^2^ = 1642.17, χ^2^/df = 4.05, CFI = 0.68, TLI = 0.63, RMSEA = 0.13. The five-factor model aiming to evaluate the validity of the five domains also showed poor goodness of fit (χ^2^ = 1561.97, χ^2^/df = 3.95, CFI = 0.70, TLI = 0.64, RMSEA = 0.13).

Model fit statistics for the respective CFA models are summarized in Table 6. Variance explained by the factors for each CFA model are presented in Table 7. To eliminate lack of power or collateral bias between set A and B as potential cause for finding the current results, we have repeated the same analyses on the other half of the data set. These analyses yielded equivalent results, supporting the robustness of our findings.

**Table 6:** Model fit statistic for the respective CFA models.

	***χ^2^***	**χ^2^/df**	**CFI**	**TLI**	**RMSEA**
Three-component model	1545.78	3.85	0.70	0.66	0.13
Three-component model, revised	1640.05	5.05	0.73	0.70	0.09
One-component model	1642.17	4.05	0.68	0.63	0.13
Five-component model	1561.97	3.95	0.70	0.64	0.13

*CFA, Confirmatory Factor Analysis; χ^2^, model-Chi-squared; df, degrees of freedom; CFI, Comparative Fit Index; TLI, Tucker Lewis Index; RMSEA, Root Mean Square Error of Approximation*.

**Table 7:** Variance explained by each factor for the respective CFA models.

	**F_**1**_**	**F_**2**_**	**F_**3**_**	**F_**4**_**	**F_**5**_**
Three-component model	0.30	0.32	0.14		
Three-component model, revised	0.47	0.67	0.72		
One-component model	0.35				
Five-component model^a^	0.51	0.42	0.73	0.54	0.44

*CFA, Confirmatory Factor Analysis; F, factor. ^a^Five-component model: F_1_ Information, F_2_ Organization, F_3_ Parental Participation, F_4_ Care and Cure, F_5_ Professional Attitude*.

## Discussion

In this study, we evaluated the psychometric characteristics of the German version of the EMPATHIC-30 for use in intermediary/general pediatric cardiology units. Furthermore, we extended the psychometric assessment in comparison to previous studies by evaluating the internal structure of the questionnaire in this care setting.

On average, parents gave high ratings for their satisfaction with FCC. The McDonald's omega values in our study indicated acceptable to good reliability for the items within the five domains and excellent reliability for the full-scale score. These values are consistent with the findings of other EMPATHIC-30 studies (26–29). We found adequate convergent validity as shown by moderate to strong correlations between the five domains scores/ the full-scale score and the four general satisfaction items. Our results fall in line with previous publications, reporting correlation coefficients in the same order of magnitude (23, 27, 28). Future studies should extend these findings by investigating convergent validity based on methodology that is more elaborate, such as the use of other standardized instruments measuring parent satisfaction with care, as well as by incorporating assessments of discriminant validity.

We used PCA to assess the internal structure of the German version of the EMPATHIC-30. The analyses from the first PCA revealed a three-component structure with an explained variance over 50%. The first component explains beyond 40%, which supports the unidimensionality of the scale and may indicate that the questionnaire adequately measures the construct of interest (satisfaction with FCC) in our population. The three-component structure resulting from the first PCA (conducted on complete case data and allowing for intercorrelations between components) is very similar to the three-component structure resulting from the second PCA (conducted on imputed data, ignoring intercorrelations between components): only two out of 30 items load differently. Considering that the correlations among the components were close to negligible in the first PCA, rotation seems to have a minor impact on the interpretation of the internal structure, which may not be true for missing data (37). Therefore, we are inclined to view the three-component structure resulting from the second PCA as the best approximation of the questionnaire's internal structure in our sample. Although the three-component solution differs from the expected five-component structure, it is plausible and interpretable. Based on the semantic content of the respective items, we label the first component as “Perception and respect of the family's needs,” the second component as “Involvement of and collaboration with the parents,” and the third as “Communication and organization.” However, despite the interpretability of the three components, the cross-validation of the three-component solutions via CFA resulted in poor fit indices. Model revisions did not significantly improve the model fit. A one-component solution to test for unidimensionality also showed a poor fit to the real data. Although the first component captures over 40% of the total variance in PCA, the true score variance seems to be relatively small compared to the random error variance. Additionally, we validated the five-component solution based on the original domains of the EMPATHIC-30, which indicated a poor fit to the real data. According to the poor model fit indices, all tested component models seem to be an oversimplification of the true structure of the questionnaire.

Our findings suggest that the EMPATHIC-30 has no clear and generalizable factor structure in our population. The ambiguous internal structure found in our study needs to be interpreted in light of the construction of the EMPATHIC questionnaires. In the original publication of the EMPATHIC-65, the five domains were defined during expert group sessions and item groupings into the respective domains were performed consensus based (24). While the authors used CFA to evaluate the unidimensionality of each domain (assessing whether the items within every domain measured the same construct), they did not evaluate the underlying factor structure of the questionnaire (23). For the development of the shortened EMPATHIC-30 questionnaire, multiple regression analysis was used to evaluate statistical performance of the items, which might explain the divergence between the conceptual and the data-driven structure of the questionnaire (26). Furthermore, scores on the EMPATHIC-30 were high on average, with relatively small standard deviations. Accordingly, the parents in our sample were highly satisfied and the limited variation may contribute to the unclear factor structure. Still, our data showed sufficient true score variation to find three interpretable dimensions. The non-zero but not very high correlations between domain scores support this claim rather than support a true unidimensional structure. Replication studies may shed light on the question whether the unclear factor structure is sample specific. For instance, individual characteristics may influence interpretation of the items and subsequently, the way items divide into latent factors. Investigating the data-driven internal structure vs. theoretically postulated structure by conducting studies in different cultural settings and (sub-) populations may therefore be an interesting avenue for follow-up research. While we did not find strong support for the five-factor structure, we consider the domains informative, especially as they were thoroughly developed through expert panels. Nevertheless, FCC reflects a multi-faceted construct and we need more conceptual work to explain expert consensus on the one hand, and unclear factorial structure on the other, especially in light of the fact that the questionnaire assesses the subjective experience, as opposed to objective criteria for FCC.

Our study warrants some limitations. This is an analysis of quality management data from a single intermediary/general pediatric cardiology unit. Participation of other pediatric cardiology centers would allow for a more robust interpretation of results and in a prospective study design, additional measurements should be included for psychometric evaluation, specifically allowing for an assessment of discriminant validity. Furthermore, based on our results, differential analyses considering population characteristics like age range, duration of stay, and complexity of disease may be important to further increase our insights into the internal structure of the questionnaire.

To sum up, the German EMPATHIC-30 has no clear and simple factor structure in our population, while showing adequate reliability and convergent validity as assessed with four general satisfaction items. Accordingly, the EMPATHIC-30 is a suitable instrument to measure FCC in intermediary/general pediatric cardiology wards. However, follow-up studies are needed to further investigate the factor structure of the questionnaire. To our knowledge, this is the first study to assess psychometric properties of a standardized assessment of satisfaction with FCC in this population. Identifying care aspects that need to be improved during hospitalization is crucial in order to meet the developmental needs of children with CHD.

## Data Availability Statement

The raw data supporting the conclusions of this article will be made available by the authors, without undue reservation.

## Ethics Statement

The studies involving human participants were reviewed and approved by the Medical Ethics Committee Charité Virchow (Nr EA2/032/20). Written informed consent for participation was not required for this study in accordance with the national legislation and the institutional requirements.

## Author Contributions

HF, KS, JL, RR, and AG contributed to the conception of the study. HF, RR, and AG developed the data analysis plan. MJ and AG prepared the dataset. RR and AG performed the statistical analysis. AG wrote the first draft of the manuscript. HF and RR reviewed the first version and wrote sections of the manuscript. All the authors contributed to the article and approved the submitted version.

## Funding

This study was partially funded by the Stiftung KinderHerz (2511–3–00–022). AG was supported by a doctoral research scholarship of the Deutsche Herzstiftung e.V. Both sponsors were not involved in the study design, data collection, analysis, interpretation, or decision to submit results.

## Conflict of Interest

The authors declare that the research was conducted in the absence of any commercial or financial relationships that could be construed as a potential conflict of interest.

## Publisher's Note

All claims expressed in this article are solely those of the authors and do not necessarily represent those of their affiliated organizations, or those of the publisher, the editors and the reviewers. Any product that may be evaluated in this article, or claim that may be made by its manufacturer, is not guaranteed or endorsed by the publisher.
