# Supplementary material for: The German EMPATHIC-30 Questionnaire Showed Reliability and Convergent Validity for Use in an Intermediary/General Pediatric Cardiology Unit: A Psychometric Evaluation
Source: Front Cardiovasc Med. 2022 Jun 23;9:901260. doi: 10.3389/fcvm.2022.901260 (PMC9262329; doi:10.3389/fcvm.2022.901260)
Supplement: Supplementary file 1 [file Data_Sheet_1.PDF]

Teil 1 Allgemeines

Fragebogen ausgefüllt am: ☐☐ ☐☐ ☐☐☐☐

Tag Monat Jahr

Ausgefüllt von:	☐ Vater<br>☐ Mutter<br>☐ Vater und Mutter<br>☐ Andere Angehörige, und zwar (z.B.: Großvater, Tante etc.):<br>…………………………………………………………
Ist Ihr Kind zum ersten Mal auf unserer Station?	☐ Ja<br>☐ Nein
Wie alt ist Ihr Kind?	☐☐ Jahre ☐☐ Monate ☐☐ Tage
Seit wie vielen Wochen und Tagen ist Ihr Kind zum momentanen Zeitpunkt stationär bei uns?	☐☐ Wochen ☐☐ Tage
War die Aufnahme Ihres Kindes in unserer Abteilung	☐ Nicht geplant/ unerwartet?<br>☐ Geplant?
Welche medizinischen Maßnahmen wurden während des Aufenthaltes durchgeführt? Mehrfachnennungen möglich!	☐ Herzkatheter-Untersuchung<br>☐ Herzoperation<br>☐ Medikamenteneinstellung oder –umstellung<br>☐ Andere, nämlich: ………………………………………………..<br>……………………………………………………………………….
Wo sind sie geboren?	☐ In Deutschland<br>☐ In einem anderem Land, nämlich:……………………………...
Was ist Ihre Muttersprache?	☐ Deutsch<br>☐ Andere Muttersprache, nämlich:………………………….….

Bitte teilen Sie uns mit, wo Sie während des stationären Aufenthaltes Ihres Kindes übernachtet haben! Mehrfachnennungen möglich!		Anzahl Nächte jeweils:
	☐ Im Zimmer des Kindes (Liege)	……Nächte
	☐ Im Zimmer des Kindes (Stuhl)	……Nächte
	☐ Auf Station (Eltern-Oase)	……Nächte
	☐ In einer durch das Krankenhaus organisierten Unterkunft (z.B. Ronald Mc Donald Haus)	……Nächte
	☐ zu Hause	……Nächte
	☐ Woanders, nämlich:<br>………....………...………..……	……Nächte

Teil 2 Ihre Erfahrungen

	Trifft gar nicht zu					Trifft voll zu	Nicht relevant
Bei der Ankunft in der Abteilung fühlten wir uns gut betreut.	☐	☐	☐	☐	☐	☐	☐
Der Arzt/die Ärztin hat uns die Folgen der Erkrankung/ Behandlung unseres Kindes deutlich erklärt.	☐	☐	☐	☐	☐	☐	☐
Wir erhielten verständliche Informationen über die Untersuchungen und Eingriffe.	☐	☐	☐	☐	☐	☐	☐
Wir erhielten verständliche Informationen über die Wirkung der Medikamente.	☐	☐	☐	☐	☐	☐	☐
Die Ärzte/ Ärztinnen und Pflegekräfte arbeiteten gut zusammen.	☐	☐	☐	☐	☐	☐	☐
Das Team achtete darauf, dass unser Kind möglichst keine Schmerzen hatte.	☐	☐	☐	☐	☐	☐	☐
Wir wurden aktiv in die Entscheidungsprozesse über die Pflege und Behandlung unseres Kindes einbezogen.	☐	☐	☐	☐	☐	☐	☐
Wir wurden ermuntert, nahe bei unserem Kind zu sein.	☐	☐	☐	☐	☐	☐	☐
Während allen Behandlungen konnten wir immer nahe bei unserem Kind bleiben.	☐	☐	☐	☐	☐	☐	☐
Das Team achtete auf die Einhaltung der Hygienevorschriften bei sich und bei den Besuchern.	☐	☐	☐	☐	☐	☐	☐
Das Team sorgte gut für die Privatsphäre unseres Kindes und von uns.	☐	☐	☐	☐	☐	☐	☐
Die Station war sauber.	☐	☐	☐	☐	☐	☐	☐
Die Station war telefonisch gut erreichbar.	☐	☐	☐	☐	☐	☐	☐
Der Lärm auf der Station war im Rahmen der Möglichkeiten gedämpft.	☐	☐	☐	☐	☐	☐	☐
Um das Bett unseres Kindes herum war genügend Platz.	☐	☐	☐	☐	☐	☐	☐
Das Team arbeitete effizient (d.h. es koordinierte die Maßnahmen, damit unser Kind beispielsweise nicht zweimal geweckt werden musste).	☐	☐	☐	☐	☐	☐	☐
Das Team zeigte Respekt gegenüber unserem Kind und uns selbst.	☐	☐	☐	☐	☐	☐	☐
Während unseres Aufenthaltes wurden wir regelmäßig nach unseren Erfahrungen und Problemen, mit denen wir konfrontiert waren, gefragt.	☐	☐	☐	☐	☐	☐	☐
	Trifft gar nicht zu					Trifft voll zu	Nicht relevant

	Trifft gar nicht zu					Trifft voll zu	Nicht relevant
Wir hatten täglich ein Gespräch über die Pflege und Behandlung unseres Kindes mit den:							
• Ärzten/Ärztinnen	☐	☐	☐	☐	☐	☐	☐
• Pflegekräften	☐	☐	☐	☐	☐	☐	☐
Auf das Wohlbefinden unseres Kindes wurde besonders geachtet von den:							
• Ärzten/Ärztinnen	☐	☐	☐	☐	☐	☐	☐
• Pflegekräften	☐	☐	☐	☐	☐	☐	☐
Wir wussten jeden Tag, wer für unser Kind verantwortlich war von den:							
• Ärzten/Ärztinnen	☐	☐	☐	☐	☐	☐	☐
• Pflegekräften	☐	☐	☐	☐	☐	☐	☐
Wir hatten Vertrauen in die:							
• Ärzte/Ärztinnen	☐	☐	☐	☐	☐	☐	☐
• Pflegekräfte	☐	☐	☐	☐	☐	☐	☐
Einfühlsam im Umgang mit uns waren die:							
• Ärzte/Ärztinnen	☐	☐	☐	☐	☐	☐	☐
• Pflegekräfte	☐	☐	☐	☐	☐	☐	☐
Wir waren gut vorbereitet auf die Verlegung auf eine andere Station oder Entlassung unseres Kindes nach Hause durch die:							
• Ärzte/Ärztinnen	☐	☐	☐	☐	☐	☐	☐
• Pflegekräfte	☐	☐	☐	☐	☐	☐	☐
	**Trifft gar nicht zu**					**Trifft voll zu**	**Nicht relevant**

Allgemeine Erfahrung

	Trifft gar nicht zu					Trifft voll zu	Nicht relevant
Wir würden diese Station weiterempfehlen.	☐	☐	☐	☐	☐	☐	☐
Wenn wir wieder in dieselbe Situation kommen, dann möchten wir gerne wieder auf diese Station.	☐	☐	☐	☐	☐	☐	☐

Wie würden Sie uns insgesamt bewerten?

Sehr schlecht	1	2	3	4	5	6	7	8	9	10	Ausgezeichnet
• Ärzte	☐	☐	☐	☐	☐	☐	☐	☐	☐	☐	
• Pflegekräfte	☐	☐	☐	☐	☐	☐	☐	☐	☐	☐	

Gerne möchten wir aus Ihrer Erfahrung lernen. Bitte nutzen Sie den nachfolgenden Raum, um uns Ihre Erfahrungen mitzuteilen.

Ihre Erfahrungen über die AUFNAHME

Ihre Erfahrungen über den AUFENTHALT

Ihre Erfahrungen über die VERLEGUNG oder ENTLASSUNG

Ihre ALLGEMEINEN Erfahrungen
