# Supplementary material for: The German EMPATHIC-30 Questionnaire Showed Reliability and Convergent Validity for Use in an Intermediary/General Pediatric Cardiology Unit: A Psychometric Evaluation
Source: Front Cardiovasc Med. 2022 Jun 23;9:901260. doi: 10.3389/fcvm.2022.901260 (PMC9262329; doi:10.3389/fcvm.2022.901260)
Supplement: Supplementary file 2 [file Table_1.DOCX]

**Supplementary Table. Comparison of component loadings after oblique and orthogonal rotation**

| **Items** | **Obliquely rotated**  **components** | | |  | **Orthogonally rotated**  **components** | | |
| --- | --- | --- | --- | --- | --- | --- | --- |
|  | **1** | **2** | **3** |  | **1** | **2** | **3** |
| Parental Participation - Confidence in nurse | 0.83 |  |  |  | 0.78 |  |  |
| Parental Participation - Confidence in doctor | 0.79 |  |  |  | 0.72 | 0.39 |  |
| Care and Cure - Child comfort nurse | 0.77 |  | 0.31 |  | 0.78 |  |  |
| Care and Cure - Child comfort doctor | 0.75 |  |  |  | 0.75 |  |  |
| Professional Attitude - Respect | 0.74 |  |  |  | 0.72 | 0.37 |  |
| Care and Cure - Teamwork | 0.73 |  |  |  | 0.70 | 0.42 |  |
| Professional Attitude - Sympathy doctor | 0.72 |  |  |  | 0.72 | 0.31 |  |
| Care and Cure - Pain treatment | 0.71 |  |  |  | 0.69 |  |  |
| Information - Disease treatment | 0.62 |  |  |  | 0.55 | 0.37 |  |
| Professional Attitude - Sympathy nurse | 0.61 |  | 0.36 |  | 0.67 |  | 0.42 |
| Professional Attitude - Admission | **0.54** | **0.48** |  |  | **0.51** | **0.59** |  |
| Professional Attitude - Hygiene | 0.49 |  | 0.34 |  | 0.55 |  | 0.38 |
| Organization - Efficiency | **0.39** | **0.36** |  |  | **0.46** | **0.49** |  |
| Parental Participation - Encouraged to stay close |  | 0.69 |  |  |  | 0.64 |  |
| Professional Attitude - Privacy |  | 0.63 |  |  |  | 0.69 |  |
| Organization - Space |  | 0.62 | 0.40 |  |  | 0.62 | 0.38 |
| Information - Drugs |  | 0.60 |  |  |  | 0.65 |  |
| Parental Participation - Asked about experiences |  | 0.59 |  |  | 0.32 | 0.66 |  |
| Parental Participation - Stay close |  | 0.54 |  |  |  | 0.61 |  |
| Parental Participation - Decision-making | 0.50 | 0.53 |  |  | 0.50 | 0.64 |  |
| Information - Examination | 0.36 | 0.44 |  |  | 0.33 | 0.56 |  |
| Organization - Noise |  | 0.43 | 0.40 |  |  | 0.43 | 0.33 |
| Care and Cure - Discharge nurse |  |  | 0.70 |  |  |  | 0.70 |
| Care and Cure - Discharge doctor |  |  | 0.67 |  |  |  | 0.70 |
| Care and Cure - Responsible nurse |  |  | 0.67 |  |  |  | 0.67 |
| Information - Daily talks with nurse | 0.44 |  | 0.54 |  | 0.54 |  | 0.58 |
| Organization - Reachable |  |  | 0.53 |  | 0.41 |  | 0.52 |
| Organization - Clean |  |  | 0.50 |  | 0.43 |  | 0.45 |
| Information - Daily talks with doctor | 0.35 |  | 0.45 |  | 0.44 |  | 0.50 |
| Care and Cure - Responsible doctor |  |  | 0.43 |  |  | 0.36 | 0.53 |

Oblique rotation based on complete case data (number of selected cases varies because of missing data).
Orthogonal rotation based on imputed data, combined component loadings (n=178).
